# Clinico-Laboratory Profile of Hypertriglyceridemia Thalassemia Syndrome: A Case Series in a Paediatric Tertiary Care Centre

**DOI:** 10.7759/cureus.67936

**Published:** 2024-08-27

**Authors:** Aritra Kapat, Raghunath Murmu, Satyajit Mandal, Koushik Biswas, Subhajit Bhakta, Asok Kumar Mandal

**Affiliations:** 1 Paediatric Medicine, Dr B C Roy Post Graduate Institute of Paediatric Sciences, Kolkata, IND; 2 Paediatric Medicine, Midnapore Medical College and Hospital, Midnapore, IND; 3 Biochemistry, All India Institute of Medical Sciences, Rae Bareli, Rae Bareli, IND

**Keywords:** primary iron overload, anemia, exome sequencing, paediatric, triglyceride, oxidative stress, hypertriglyceridemia thalassemia syndrome, hypertriglyceridemia (htg), lipid profiles, thalassemia

## Abstract

Background: Increased hemolysis and repeated blood transfusion trigger oxidative stress resulting in numerous adverse effects in beta-thalassemia patients. Extreme elevation of triglyceride level is a rare clinical entity seen in these patients. It is presumed to be caused due to an increase in oxidative stress and is termed Hypertriglyceridemia Thalassemia Syndrome.

Objectives: To assess the clinical and laboratory characteristics of beta-thalassemia patients presenting with hypertriglyceridemia and its correlation with the pre-transfusion hemoglobin level.

Methods: This hospital record-based retrospective study was conducted at the Dr B C Roy Post Graduate Institute of Paediatric Sciences, Kolkata, India. The study comprised 12 pediatric beta-thalassemia patients whose plasma appeared milky or chylous during a complete hemogram. Clinical examination and laboratory investigations were done to describe their clinico-laboratory features. A whole exome sequencing was carried out to assess their genetic background. Blood hemoglobin and serum triglyceride estimation was carried out initially and at follow-up to determine any correlation between the two.

Results: Out of 1482 patients, 12 (0.80 %) were diagnosed with Hypertriglyceridemia Thalassemia Syndrome. The median age of presentation was 12.5 months (Q1:10 months, Q3:14 months)., and the pretransfusion hemoglobin was 4.82 ± 1.16 g/dL. The lipid profile showed a triglyceride level of 858.3 ± 198.4 mg/dl and a total cholesterol level of 117.4 ± 16.15 mg/dl. Analysis revealed that the triglyceride levels were negatively correlated with the pretransfusion hemoglobin level (repeated measures correlation (rmcorr) = -0.65, 95% CI [-0.794, -0.425], p < 0.001). A genetic study highlighted c.92+5G>C as the commonest mutation.

Conclusion: Hypertriglyceridemia was a rare presentation in transfusion-dependent beta-thalassemia patients. The serum triglyceride level significantly reduced when blood transfusion at regular intervals restored the patient's hemoglobin level.

## Introduction

Beta-thalassemia is a group of chronic hereditary hematological disorders characterized by abnormal beta-chain synthesis of hemoglobin, with phenotypic variations ranging from clinically asymptomatic to severe hemolytic anemia. It is present globally and highly prevalent in Southeast Asia, South China, the Middle East, and Mediterranean countries [1]. Depending on the patient’s genetic analysis, β-thalassemia patients are classified into β-thalassemia major, β-thalassemia intermedia, or β-thalassemia Minor (carriers). β-thalassemia Major (BTM) patients require regular lifelong blood transfusion along with adequate iron chelation therapy, whereas β-thalassemia Intermedia (BTI) patients might require no or occasional blood transfusion [2-3]. In clinical practice, these patients are categorized into Transfusion-dependent-β-thalassemia (TDT) and Non-transfusion-dependent-β-thalassemia (NTDT) [3]. 

Globally, many patients with BTM have limited access to regular and safe blood transfusions. In resource-constrained countries, fewer voluntary non-remunerated blood donors, poor public awareness, lack of national blood donation policy, and fragmented blood transfusion service create a significant gap between the timely supply and demand of safe blood [4]. Regular blood transfusions are associated with multiple adverse effects like iron overload, transfusion-related infections, and transfusion reactions due to alloimmunization [5]. Oxidative stress in these patients results in cytotoxicity and organ damage. The symptoms aggravated by oxidative stress include increased hemolysis, ineffective erythropoiesis, metabolic derangements, and functional failure of vital organs such as the heart and liver. The oxidative status of a beta-thalassemia patient depends on multiple factors such as genetic predisposition, nutrition, age, environment, variability of transfusion strategy, as well as the clinical condition *viz.* unpaired globin chains, severity of pre-transfusion anemia, and iron overload [6].

Boudrahem-Addour et al. [7] reported that increased oxidative stress (as estimated by malondialdehyde, reduced glutathione, and catalase activity) adversely affected lipid profiles in BTM patients. Different studies analysing the lipid profile in thalassemia patients highlight a lower serum total cholesterol, LDL cholesterol, and HDL cholesterol level with a mild elevation in serum triglyceride level [8-9]. However, a very high triglyceride level is a rare phenomenon in thalassemia patients. This entity is known as Hypertriglyceridemia Thalassemia Syndrome (HTS) and has an unclear pathogenesis. It is particularly seen in pediatric thalassemia patients, and it is important to detect early for the prevention of complications [10-11]. As limited information exists on this pathology, this case series emphasises on describing the clinical and laboratory characteristics of patients with Hypertriglyceridemia Thalassemia Syndrome (HTS).

## Materials and methods

Study setting and design

This study was conducted at the Dr B C Roy Post Graduate Institute of Paediatric Sciences, Kolkata, India, a tertiary care paediatric center in eastern India. The data of beta-thalassemia patients admitted to the institute for blood transfusion from April 2021 to March 2024, was retrieved from the hospital records. The data was analysed over the next three months. This is a descriptive case series.

Inclusion and exclusion criteria

The diagnosis of thalassemia was confirmed by hemoglobin (Hb) high-pressure liquid chromatography (HPLC) test. Patients with transfusion-dependent thalassemia admitted to the thalassemia special clinic for routine packed red blood cells (PRBC) during the study duration were included. Undiagnosed cases of chronic hemolytic anaemia were excluded.

Study procedure

**Figure 1:**
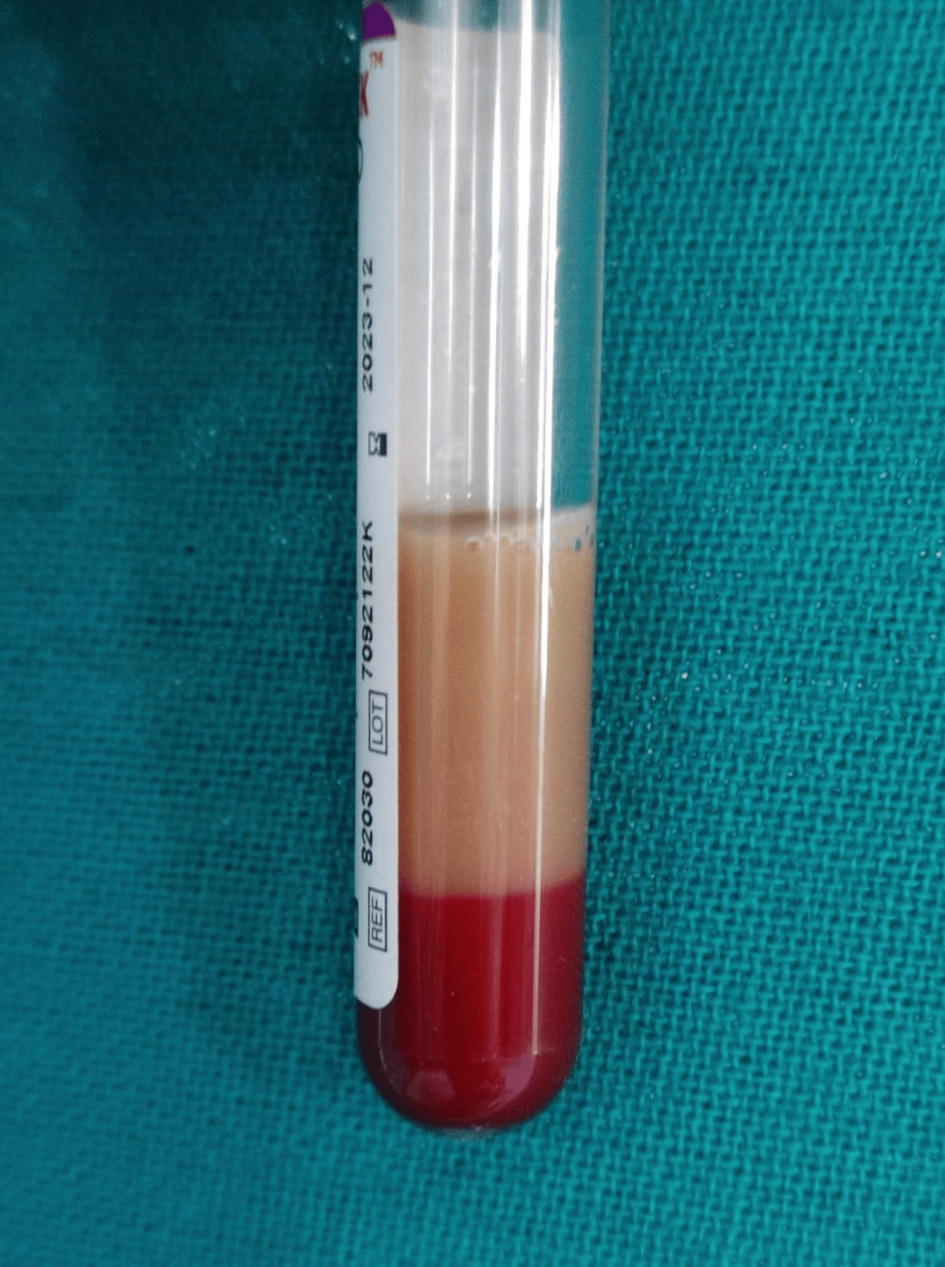
Milky or chylous-appearing plasma A representative sample.

All transfusion-dependent beta-thalassemia patients attending the thalassemia special clinic were advised to get a complete hemogram as routine practice. During sample collection, when the blood was collected and allowed to stand for 30 minutes, it was observed that the plasma of a few patients was milky or chylous in appearance (Figure 1).

These patients were suspected to have dyslipidemia. They underwent further investigations like liver function tests, renal function tests, lipid profile, and routine urine examinations. Serum ferritin was estimated for patients who had a history of over ten episodes of blood transfusion. The lipid profile of these patients was re-checked on subsequent hospital visits. The lipid profile of both parents of all these patients was evaluated to detect familial dyslipidemia. Denver II developmental screening test was used to screen developmental milestones in patients. The genetic background of these patients was conducted via multiplex amplification refractory mutation system-induced polymerase chain reaction (ARMS-PCR). Their lipid profile and complete hemogram were again evaluated at a follow-up visit after a period of one year, by which time the patient had received two to five episodes of regular transfusion.

Statistical analysis

Patient data was recorded in a pre-designed case record form and entered into an Excel spreadsheet (Microsoft Inc., Redmond, Washington, USA). Results of continuous variables were presented as mean ± standard deviation for parametric data and median with interquartile range for nonparametric data. Results from categorical variables were presented in frequency and percentage. Repeated Measures Correlation (rmcorr) was applied between serial measurements of pre-transfusion hemoglobin and serial triglyceride, as described by Marusich and Bakdash [12].

Ethical approval

The study was approved by the Institutional Ethics Committee (Memo No. BCH/ME/PR/427A dated 12.03.2024). It was conducted as per regulations mentioned in the Helsinki Declaration of 1964, as revised in 2013 and the local regulations.

## Results

During our study duration 1482 beta-thalassemia patients attending the thalassemia special clinic were admitted for packed cell transfusion. Out of them only 14 (0.94 %) were found to have chylous or milky plasma. Of these 14 patients, two were diagnosed with familial hypertriglyceridemia, where either or both parents were found to have elevated triglyceride levels. The remaining 12 (0.80 %) patients were suspected of Hypertriglyceridemia Thalassemia Syndrome and were further analysed.

These 12 patients included seven (58.3 %) male and five (41.7 %) female patients. The median age of presentation was 12.5 months (Q1:10 months, Q3:14 months). The pretransfusion hemoglobin was 4.82 ± 1.16 g/dL. Among these 12 patients, seven (58.3%) had hepatosplenomegaly, whereas four (33.3 %) had only splenomegaly. Three patients (25%) were clinically detected with jaundice on their first presentation. A delay in motor milestones was observed in three (25%) out of 12 patients. The clinical features and hematological parameters of these 12 patients are presented in Table 1. Hemoglobin HPLC reports revealed that of these 12 patients, five (41.6%) had E-β-thalassemia while seven (58.4%) had β-thalassemia. The genetic profile highlighted that c.92+5G>C mutation was present in eight (66.7 %) patients, whereas c.79G>A mutation was present in five (41.7 %) patients. The other mutations detected in patients were c.92G>C and CD41/CD42(-CTTT) (Table 1).

**Table 1:** Clinical Features and Hematological Parameters M: Male, F: Female, Hb: Hemoglobin, g/dl: gram per deciliter, MCV: Mean Corpuscular Volume, fl: femtolitre; MCH: Mean Corpuscular Hemoglobin, pg: picogram, HbF: hemoglobin F, HbA: hemoglobin A, HbA2: hemoglobin A2; HbE+A2: Hemoglobin E+A2

Patient no.	Age (in months) / Sex	Clinical features	Hb (in g/dl)	MCV (in fl) /MCH (in pg)	HbF%	HbA%	HbA2%	HbE+A2%	Genetics
1	6/M	Pallor, Poor Weight gain	4.7	66.5/23.2	4.50	73.60	-	10.40	c.92+5G>C & c.79G>A
2	9/M	Pallor, splenomegaly, Poor weight gain	6.1	58/16.4	90.40	-	4.50	-	c.92G>C (p.Arg31Thr )
3	24/F	Pallor, Jaundice, hepato-splenomegaly	5.7	58.2/17.6	84.50	9.70	6.10	-	c.92+5G>C
4	14/M	Pallor, hepato-splenomegaly	4.6	75.5/16.7	32.50	-	-	60.60	CD41/CD42(-CTTT)
5	11/F	Pallor, hepato-splenomegaly	2.9	82.5/26.7	21.50	-	2.30	-	c.92+5G>C & c.79G>A
6	13/F	Pallor, splenomegaly	5.5	66.4/20.0	36.50	5.50	-	59.70	CD41/CD42(-CTTT)
7	14/M	Pallor, motor delay, hepato-splenomegaly	5.9	51.6/19.5	28.70	-	-	64.40	c.92+5G>C & c.79G>A
8	14/M	Pallor, poor growth, hepato-splenomegaly	4.1	49.1/20.0	36.00	-	-	46.30	c.92+5G>C & c.79G>A
9	19/M	Pallor, Jaundice, massive splenomegaly, hepatomegaly	3.3	56/18.8	90.90	2	3.40	-	c.92+5G>C & c.79G>A
10	11/M	Pallor, Jaundice, hepatomegaly	3.1	54/19.9	26.90	-	3.20	-	c.92+5G>C
11	12/F	Pallor, motor delay, poor growth, splenomegaly	6.0	62.5/22.7	74.40	8.10	2.20	-	c.92+5G>C
12	8/F	Pallor, splenomegaly	5.9	60.5/21.3	41.00	-	2.90	-	CD41/CD42(-CTTT)

Lipid profile analysis of these 12 patients revealed elevated serum triglyceride levels. However, their serum total cholesterol was within the normal reference range. The lipid profile showed a triglyceride level of 858.3 ± 198.4 mg/dl and a total cholesterol level of 117.4 ± 16.15 mg/dl. The total cholesterol and triglyceride of both parents were also normal (Table 2). The serum urea and creatinine of all patients were within the normal reference range. The serum ferritin level of the patients (n=9) was 285.44 ± 127.26 ng/ml. 

**Table 2:** Lipid profile of patients and their both parents LDL-C: low-density lipoprotein cholesterol, mg/dl: milligram per decilitre

Patient no.	Triglyceride (in mg/dl)	Total Cholesterol (in mg/dl)	LDL-C (in mg/dl)	Father’s Triglyceride (in mg/dl) / Total Cholesterol (in mg/dl)	Mother’s Triglyceride (in mg/dl) /Total Cholesterol (in mg/dl)
1	890	130	80	95/189	112/90
2	750	125	46	102/204	106/95
3	850	150	58	106/165	105/85
4	1150	110	47	100/85	95/123
5	660	100	66	132/140	125/140
6	1200	130	110	95/95	104/122
7	980	90	40	85/100	65/115
8	1040	95	45	80/86	78/90
9	880	125	52	110/154	102/85
10	650	116	90	75/165	49/124
11	720	122	48	85/65	85/100
12	530	116	50	65/135	90/80

**Figure 2:**
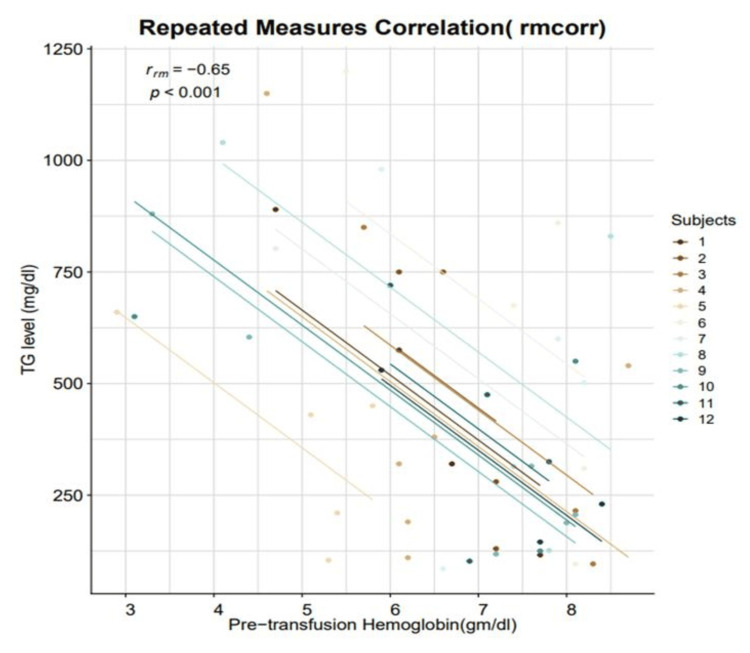
Correlation between serum triglyceride and pre-transfusion hemoglobin level r_rm_: repeated measures correlation coefficient, p: p-value, TG: triglyceride, mg: milligram, g: gram, dl: decilitre

On analysing the serum triglyceride levels at the initial visit and at follow-up after two to five episodes of transfusion under supervision, we observed that the serum triglyceride level had a negative correlation with the pre-transfusion hemoglobin level (rmcorr = -0.65, 95% CI [-0.794, -0.425], p < 0.001) (Figure 2). At follow-up after one year, patients had normal serum amylase and lipase levels. No atherosclerotic changes were observed in the bilateral carotid intimal medial thickness of any patient.

## Discussion

Patients with transfusion-dependent thalassemia often show many inevitable biophysical and biochemical changes, iron overload being a significant causative factor. Growth failure, hypersplenism, chronic hemolysis, impaired immunity, hypothyroidism, hypoparathyroidism, hypogonadism, delayed puberty, osteopenia, impaired glucose tolerance, propensity for cardiomyopathies, cerebrovascular accidents, and liver failure are all part of diverse organ and metabolic disorders [13]. The unusual alliance of hypertriglyceridemia and thalassemia is described as a rare entity, according to some authors, and is given the categorical nomenclature “hypertriglyceridemia thalassemia syndrome" [10].

Hypertriglyceridemia implies a rise in plasma fasting triglyceride (TG) levels above the 95th percentile as per age and sex. For children of age groups 0 to 9 years and 10 to 19 years, a fasting serum TG concentration ≥ 100 mg/dL (1.13 mmol/L) and a concentration ≥ 130 mg/dL (1.47 mmol/L) are considered above the 95th percentile, respectively, labelling them to be hypertriglyceridemia [14]. The Endocrine Society Clinical Practice Guidelines published in 2010 and the European Society Consensus Panel in 2012 opined a cut-off of more than 150 mg/dL to be hypertriglyceridemia [15-16]. Frederickson and Levy classified primary dyslipidemia on the basis of lipoprotein levels into five major groups. Among them, groups I, III, IV and V are associated with marked hypertriglyceridemia, although it is rare in the pediatric age group [10]. Rao et al. [17] first reported that hyperglycemia seen in thalassemia patients was not associated with familial dyslipidemia, tuberous xanthoma, tendon xanthoma or any other secondary risk factors. In this study, we observed a similar presentation in the 12 children adjudged to have hypertriglyceridemia thalassemia syndrome. In our study, the median age of presentation was 12.5 months. Khera et al. [10] reported the age of presentation as four months to 2.5 years in their study.

The pathogenesis of such an association is still obscure as the cases are sporadic, but a strong association with an underlying hemolytic crisis cannot be denied. In this study, the initial mean hemoglobin at the presentation was 4.82 ± 1.16 g/dL, which points towards a massive hemolytic state. A hemolytic crisis can serve as the origin of a pro-inflammatory cascade encompassing tumor necrosis factor-α (TNF-α), interferon-α, and other cytokines, which, in conjunction with oxidative stress due to malnutrition, might elicit hypertriglyceridemia [18]. Thalassemia patients are more prone to infections, and TNF-α reduces lipoprotein lipase activity in adipose tissue [19]. Other cytokines also impair hepatic very low-density lipoprotein production in response to the infectious trigger, which might also contribute to hypertriglyceridemia [20].

This early-onset hypertriglyceridemia can have a deleterious effect on the patient’s prognosis as it increases the risk of cardiovascular morbidity, acute pancreatitis, and non-alcoholic fatty liver disease [10]. Serum triglyceride levels above 1000 mg/dL prompt a search for monogenic hypertriglyceridemia. In this study, the mean triglyceride level of 12 patients was 858.3 ± 198.4 mg/dL. Among the 12 patients, only three had serum triglyceride levels above 1000 mg/dL. However, whole exome sequencing excluded any monogenic background in them. This further confirms their diagnosis as cases of hypertriglyceridemia thalassemia syndrome. Previous studies reported that repeated blood transfusions of BTM patients under supervision at regular intervals, which led to hypertransfusion, decreased ineffective erythropoiesis, suppressing the cytokine cascade, which in turn ameliorated hypertriglyceridemia [21]. Although niacin and omega-3 fatty acids are often used to lower serum triglyceride, there is a paucity of data regarding their dosage and safety in young children [14]. In this study, we observed a negative correlation between triglyceride level and pre-transfusion hemoglobin level (Figure 2). We further observed that the serum triglyceride level of all 12 patients spontaneously reduced after two to five episodes of repeated transfusions at regular intervals (Figure 2). Subsequent follow-up of these patients at one year ruled out elevation in serum amylase or lipase and atherosclerotic changes in bilateral carotid arteries.

In this study, we conducted whole-exome sequencing of the 12 patients to identify any genetic predisposition associated with hypertriglyceridemia. The most common mutation identified was c.92+5G>C, which is also found in the Mediterranean area [15-16]. To date, the correlation between the presence of a mild beta-thalassemia/severe beta-thalassemia genotype and a mild/severe clinical phenotype cannot be established due to the presence of a large number of genetic modifiers. Hence, individuals with the same β-thalassemia genotype may reflect wide phenotypic variability ranging from moderate to severe diseases with comorbidities [10].

The main advantage of this study is that it tried to explore the clinical features, laboratory parameters and genetic predisposition of patients with Hypertriglyceridemia Thalassemia Syndrome. The main limitation is its small sample size. Multi-centric research on this area with a larger sample size can help to know more about the genetic predisposition and frame a treatment strategy for this condition. Another limitation of our study was that we had restricted our analysis to samples that had chylous or milky appearing plasma. The prevalence of this syndrome might be a little higher if we analysed all samples.

## Conclusions

This case series should be of interest to paediatricians dealing with thalassemia patients. Hypertriglyceridemia Thalassemia Syndrome is a rare metabolic abnormality observed mainly in late infancy and early childhood where very high triglyceride levels are noted in poorly transfused thalassemia patients. This condition may be undiagnosed in low- and medium-income settings. Clinicians should suspect this condition if chylous or milky plasma is observed during routine phlebotomy to check the complete hemogram of thalassemia patients prior to transfusion. We observed that this elevated triglyceride level tends to normalize over time when the blood hemoglobin level is gradually restored by repeated transfusion at regular intervals under supervision. If undetected hypertriglyridemia may have adverse effects on the patient’s health in the long run.

## References

[1] Li CK (2017). New trend in the epidemiology of thalassaemia. Best Pract Res Clin Obstet Gynaecol.

[2] (2014). Guidelines for the management of transfusion dependent thalassaemia (TDT). https://pubmed.ncbi.nlm.nih.gov/25610943/.

[3] Yardumian A, Telfer P, Shah F (2016). Standards for the clinical care of children and adults with thalassaemia in the UK. https://ukts.org/wp-content/uploads/2021/02/Standards-2016final.pdf.

[4] Shah FT, Sayani F, Trompeter S, Drasar E, Piga A (2019). Challenges of blood transfusions in β-thalassemia. Blood Rev.

[5] Wolman IJ, Ortolani M (1969). Some clinical features of Cooley's anemia patients as related to transfusion schedules. Ann N Y Acad Sci.

[6] Fibach E, Dana M (2019). Oxidative stress in β-thalassemia. Mol Diagn Ther.

[7] Boudrahem-Addour N, Izem-Meziane M, Bouguerra K, Nadjem N, Zidani N, Belhani M, Djerdjouri B (2015). Oxidative status and plasma lipid profile in β-thalassemia patients. Hemoglobin.

[8] Goldfarb AW, Rachmilewitz EA, Eisenberg S (1991). Abnormal low and high density lipoproteins in homozygous beta-thalassaemia. Br J Haematol.

[9] Al-Quobaili FA, Abou Asali IE (2004). Serum levels of lipids and lipoproteins in Syrian patients with beta-thalassemia major. Saudi Med J.

[10] Khera R, Singh M, Goel G, Gupta P, Singh T, Dubey AP (2014). Hypertriglyceridemia thalassemia syndrome: a report of 4 cases. Indian J Hematol Blood Transfus.

[11] Das L, Samprathi M, Shukla U, Bandyopadhyay D, Das RR (2016). Hypertriglyceridemia thalassemia syndrome: common disease, uncommon association. Indian J Pediatr.

[12] Marusich LR, Bakdash JZ (2021). rmcorrShiny: A web and standalone application for repeated measures correlation. F1000Res.

[13] Shamshirsaz AA, Bekheirnia MR, Kamgar M (2003). Metabolic and endocrinologic complications in beta-thalassemia major: a multicenter study in Tehran. BMC Endocr Disord.

[14] Valaiyapathi B, Sunil B, Ashraf AP (2017). Approach to hypertriglyceridemia in the pediatric population. Pediatr Rev.

[15] Berglund L, Brunzell JD, Goldberg AC, Goldberg IJ, Sacks F, Murad MH, Stalenhoef AF (2012). Evaluation and treatment of hypertriglyceridemia: an Endocrine Society clinical practice guideline. J Clin Endocrinol Metab.

[16] Hegele RA, Ginsberg HN, Chapman MJ (2014). The polygenic nature of hypertriglyceridaemia: implications for definition, diagnosis, and management. Lancet Diabetes Endocrinol.

[17] Rao AV, Bai KI, Ramanujiah D (1972). Hypertriglyceridaemia in thalassaemia major. J Indian Med Assoc.

[18] Mousavi Z, Yazdani Z, Moradabadi A, Hoseinpourkasgari F, Hassanshahi G (2019). Role of some members of chemokine/cytokine network in the pathogenesis of thalassemia and sickle cell hemoglobinopathies: a mini review. Exp Hematol Oncol.

[19] Kern PA (1997). Potential role of TNFalpha and lipoprotein lipase as candidate genes for obesity. J Nutr.

[20] Fried SK, Zechner R (1989). Cachectin/tumor necrosis factor decreases human adipose tissue lipoprotein lipase mRNA levels, synthesis, and activity. J Lipid Res.

[21] Rao A, Hulbert M, Wilson DB (2007). Severe hypertriglyceridemia in an infant with red cell pyruvate kinase deficiency. Indian Pediatr.

